# Intrinsic Inflammation Is a Potential Anti-Epileptogenic Target in the Organotypic Hippocampal Slice Model

**DOI:** 10.1007/s13311-018-0607-6

**Published:** 2018-02-20

**Authors:** Seon-Ah Chong, Silvia Balosso, Catherine Vandenplas, Gregory Szczesny, Etienne Hanon, Kasper Claes, Xavier Van Damme, Bénédicte Danis, Jonathan Van Eyll, Christian Wolff, Annamaria Vezzani, Rafal M. Kaminski, Isabelle Niespodziany

**Affiliations:** 1 UCB Biopharma SPRL, Chemin du Foriest, B-1420 Braine l’Alleud, Belgium; 2 Department of Neuroscience, IRCCS-Istituto di Ricerche Farmacologiche Mario Negri, Milan, 20156 Italy

**Keywords:** Neuroinflammation, TBI, epileptogenesis, TNFα, OHSCs, MEA

## Abstract

Understanding the mechanisms of epileptogenesis is essential to develop novel drugs that could prevent or modify the disease. Neuroinflammation has been proposed as a promising target for therapeutic interventions to inhibit the epileptogenic process that evolves from traumatic brain injury. However, it remains unclear whether cytokine-related pathways, particularly TNFα signaling, have a critical role in the development of epilepsy. In this study, we investigated the role of innate inflammation in an *in vitro* model of post-traumatic epileptogenesis. We combined organotypic hippocampal slice cultures, representing an *in vitro* model of post-traumatic epilepsy, with multi-electrode array recordings to directly monitor the development of epileptiform activity and to examine the concomitant changes in cytokine release, cell death, and glial cell activation. We report that synchronized ictal- and interictal-like activities spontaneously evolve in this culture. Dynamic changes in the release of the pro-inflammatory cytokines IL-1β, TNFα, and IL-6 were observed throughout the culture period (3 to 21 days *in vitro*) with persistent activation of microglia and astrocytes. We found that neutralizing TNFα with a polyclonal antibody significantly reduced ictal discharges, and this effect lasted for 1 week after antibody washout. Neither phenytoin nor an anti-IL-6 polyclonal antibody was efficacious in inhibiting the development of epileptiform activity. Our data show a sustained effect of the anti-TNFα antibody on the ictal progression in organotypic hippocampal slice cultures supporting the critical role of inflammatory mediators in epilepsy and establishing a proof-of-principle evidence for the utility of this preparation to test the therapeutic effects of anti-inflammatory treatments.

**Electronic supplementary material:**

The online version of this article (10.1007/s13311-018-0607-6) contains supplementary material, which is available to authorized users.

## Introduction

Epilepsy is a chronic disease with recurrent and unprovoked seizures that seriously impacts on the quality of life of patients. Symptomatic (acquired) epilepsy may evolve from various injuries to the brain such as infection, stroke, tumor, traumatic brain injury (TBI), and neurodegenerative disorders [1, 2]. During epileptogenesis, the brain undergoes molecular, cellular, structural, and functional changes that often include neuronal cell death, axonal sprouting, and dysfunction of voltage-gated or receptor-operated ion channels or neurotransmitter systems [1, 3]. Classical anti-epileptic drugs (AEDs) can efficiently control seizures in up to 70% of patients, but none of them has shown convincing effects on preventing or delaying the development of epilepsy [4–6]. Considerable effort has been made in the last decade to understand the mechanisms of epileptogenesis and to identify new therapeutic targets and pathways for preventing or modifying the epileptogenic process [7–10]. Neuroinflammation, among others, has been proposed as a promising point of intervention in acquired epilepsy [11, 12]. Growing evidence from rodent and human studies suggests that excessive activation of microglia and astrocytes and the concomitant increased expression of pro-inflammatory cytokines such as interleukin-1 beta (IL-1β), tumor necrosis factor-alpha (TNFα), and interleukin-6 (IL-6) following brain insults contribute to hyperexcitability and neuronal damage [13–20]. Murashima et al. [21] reported a progressive increase of IL-1α, IL-1β, and TNFα expression during the development of epilepsy in epileptic mutant (EL) mice with secondarily generalized seizures. Interestingly, the cytokine level periodically increased prior to seizure development implying a potential role of the cytokines in triggering the ictal activity. In particular, TNFα mediates either pro- or anti-convulsive effects by differential activation of receptor type 1 (TNFR1) and type 2 (TNFR2), respectively [22–24]. TNFR1 signaling increases glutamatergic neurotransmission by upregulating calcium-permeable AMPA and NMDA receptors and by inhibiting glutamate re-uptake by astrocytes [14, 25–27] which may promote seizure generation. However, it remains unclear whether TNFα signaling has a critical role in epileptogenesis and whether blocking TNFα activity with specific antibodies inhibits this pathologic process.

Because of the potential involvement of inflammatory molecules, in particular cytokines, in seizure mechanisms, it is important to investigate their role using pharmacological approaches in a well-controlled and simplified *in vitro* system. The results obtained with such a model would provide support for subsequent *in vivo* efficacy studies. Organotypic hippocampal slice cultures (OHSCs) are considered as a post-traumatic epileptogenesis model because spontaneous epileptiform activity develops after the traumatic injury occurring during slice preparation [28, 29]. This model system has been extensively studied by K. Staley’s group and used to investigate epileptogenesis mechanisms and new therapeutic targets [29–35]. It has been well proven to manifest clinical features of TBI-induced epileptogenesis such as synaptic reorganization, axonal sprouting, post-traumatic seizures, and activity-dependent cell death [29, 36–38]. In the present proof-of-concept study, we combined OHSCs with multi-electrode array (MEA) recordings and monitored cytokine release to establish a relationship between epileptogenesis and activation of inflammatory responses. We focused our investigations on the contribution of TNFα-related pathways to epileptogenesis by examining the impact of blocking TNFα on the epileptogenic process.

## Methods

### Ethical Statement

All animals used in this study were performed according to the guidelines of the European Community Council Directive 2010/63/EU. All experimental protocols using animals were reviewed and approved by the ethical committee at UCB Biopharma.

### Organotypic Hippocampal Slice Cultures (OHSCs)

OHSCs were prepared using polydimethylsiloxane (PDMS) mini-wells as described previously [39]. Briefly, liquid mix of PDMS and the curing agent (Sylgard 184, Dow Corning, Auburn, MI) was spin-coated on a 6-in. silicon (SiO_2_) wafer at 500 rpm and cured at 115 °C for 20 min then 100 °C for 1 h 30 min. The thickness of the cured membrane was within the range of 130 to 150 μm. The membrane was cut into 1 × 1 cm^2^ pieces then a mini-well (3 mm diameter) and 2 channels were made on each piece. PDMS membranes were sterilized with 70% isopropyl alcohol and placed on MEAs or on glass coverslips precoated with poly-D-lysine (Sigma-Aldrich, St. Louis, MO).

Hippocampi were dissected from 6- to 9-day-old Sprague-Dawley rats (any sex) and cut into 350-μm-thick slices using a McIlwain tissue chopper. Slices were kept in ice-cold Gey’s balanced salt solution containing 0.6% D-glucose and 300 μM kynurenic acid (all from Sigma-Aldrich, St. Louis, MO) for 30 min. After recovery, slices were washed 3 times with the culture medium containing Neurobasal A, B27 supplement, 0.5 mM Glutamax (all from ThermoFisher Scientific, Gent, Belgium), and 30 μg/ml gentamicin (Sigma-Aldrich, St. Louis, MO). Only slices from the middle part of the hippocampus were selected and placed in the center of the mini-wells which allowed a good positioning over the MEA electrodes (200/30, 60 ITO from Multi Channel Systems, Reutlingen, Germany). For immunostaining experiments, slices were cultured on glass coverslips containing the mini-wells in a 6-well culture plate. OHSCs were maintained in a humidified CO_2_ incubator at 35 °C and the medium was refreshed 2 to 3 times a week.

Goat anti-rat TNFα polyclonal antibody (Cat.# AF-510, R&D Systems, Minneapolis, MN) was dissolved in the culture medium and applied to cultures from days 0 to 14 to the final concentration of 10 μg/ml. Goat anti-rat IL-6 polyclonal antibody (Cat.# AF-506, R&D Systems, Minneapolis, MN) was also used at the same concentration and applied to cultures from days 0 to 14. The vehicle group was treated with the normal culture medium without the antibody. Phenytoin (Sigma-Aldrich, St. Louis, MO) was dissolved in dimethyl sulfoxide (DMSO) and applied to cultures from days 0 to 14 or from days 0 to 21 to the final concentration of 50 μM; 0.1% DMSO was used as a vehicle.

### Multi-Electrode Array (MEA) Recording and Data Analysis

**Fig. 1:**
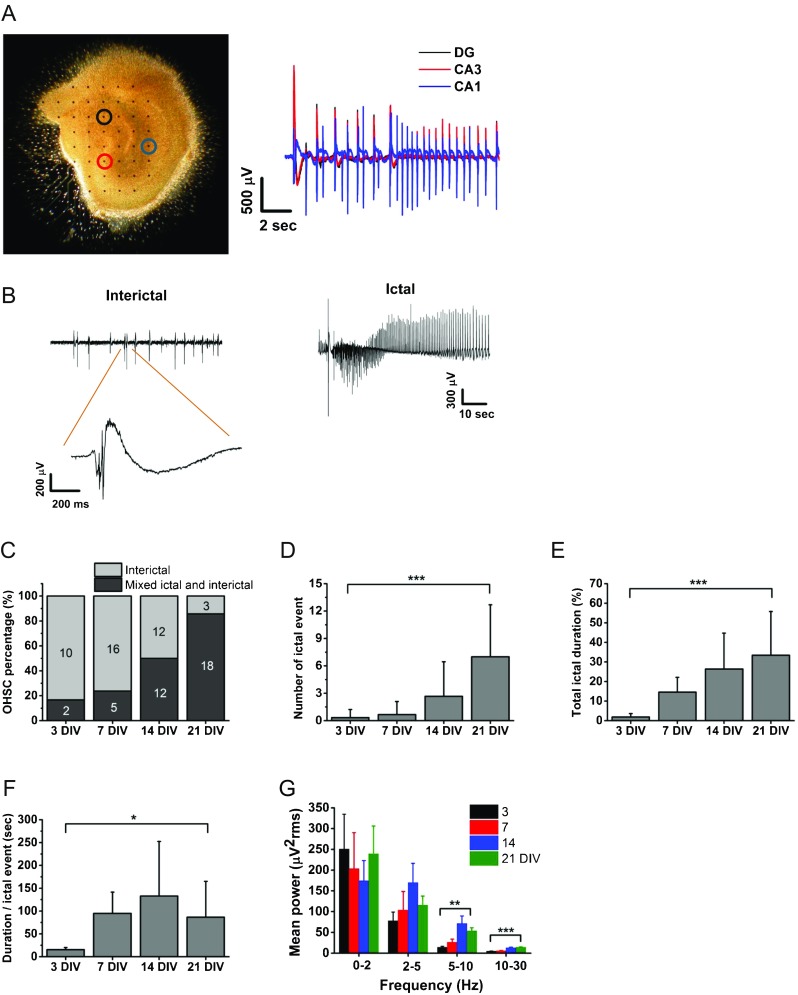
Time course of development of epileptiform activity in OHSCs. (A) Image of a hippocampal slice cultured on a MEA for 10 days *in vitro* (DIV). Epileptic signals were selected from the electrode marked as colored circles. Black, red, and blue electrodes correspond to the signals in DG, CA3, and CA1 region, respectively. (B) Representative interictal and ictal event in the CA3 region. (C) The graph shows the percentage of cultures displaying only interictal- or mixed ictal- and interictal-like activities at different DIVs. The number in the columns indicates the number of recorded slice cultures in each category. (D–F) Several parameters of epileptiform activities were analyzed from signals recorded for 30 min. Data are presented as mean ± S.D. (D) Mean number of ictal events per slice at different DIVs. Overall ***p < 0.001, pairwise comparison *p* < 0.001 for 3 *versus* 21 DIV and 7 *versus* 21 DIV, *p* = 0.001 for 14 *versus* 21 DIV. (E) Total duration of ictal events was averaged and normalized to the entire recording time (30 min). Overall ***p < 0.001, pairwise comparison *p* < 0.001 for 3 *versus* 21 DIV and 7 *versus* 21 DIV, *p* = 0.01 for 14 *versus* 21 DIV. (F) Mean duration per ictal event at different DIVs. Overall *p < 0.05. (G) Summation of power spectrum values (μV^2^ rms) by frequency range (0-< 2, 2-< 5, 5-< 10, 10-< 30 Hz). Data are presented as mean ± S.E.M. Significant differences were found for 5 to < 10 and 10 to < 30 Hz. At 5 to < 10 Hz overall **p < 0.01, pairwise comparison *p* < 0.01 for 3 *versus* 14 DIV and *p* < 0.05 for 7 *versus* 14 DIV. At 10 to < 30 Hz overall ***p < 0.001, pairwise comparison *p* < 0.001 for 3 *versus* 14, 7 *versus* 14, 3 *versus* 21, and 7 *versus* 21 DIV. Statistical differences were assessed by 1-way ANOVA followed by Tukey’s post hoc test

Extracellular recording and stimulation were performed using the MEA2100 system (2 × 60 channels, Multi Channel Systems, Reutlingen, Germany). OHSCs grown on MEAs at different days *in vitro* (DIVs) were quickly moved to the MEA recording chamber and spontaneous field activity was recorded for 40 min using MC Rack software (Multi Channel Systems, Reutlingen, Germany). The recording pads were preheated at 35 °C, and 95% O_2_/5% CO_2_ gas was continuously flowing into the chamber while recording. At the end of the recording, OHSCs showing no activity or only interictal activity were further examined for viability and synaptic response. Extracellular field potentials were evoked in the CA1, CA3, and dentate gyrus (DG) regions followed by biphasic current pulse stimulation (± 30 μA) to Schaffer collaterals, stratum lucidum, and perforant path, respectively. Only slices showing > 500 μV of peak-to-peak field excitatory post-synaptic potential (fEPSP) amplitude in each region were selected for data analysis. In the time course study (Fig. 1), 8 of 49 slices showing only interictal-like activity did not meet the quality control criteria described above, therefore were not included in the data analysis. Data was collected at 1 kHz sampling frequency. Because epileptiform activity is highly synchronized in all subregions of the hippocampus (Fig. 1), we chose 1 electrode in the CA3 pyramidal cell layer to analyze epileptic activity. Because cultured slices were required to adjust to the recording chamber, we excluded signals from the first 10 min and only the last 30 min of recorded data was used for the data analysis. We developed an in-house software using Labview (National Instruments, Austin, TX) to automatically detect ictal events. Recording files (.mcd) were downsampled to 250 Hz that is adequate to capture local field potentials [40].

Based on the previous publications describing epileptic signal analysis in acute and cultured hippocampal slices [29, 31, 41–43] and reviewing the ictal and interictal signals in our model, we have defined interictal epileptiform discharges as paroxysmal discharges that are clearly distinguished from background activity, with an abrupt change in polarity occurring at low frequency (< 2 Hz). We have defined ictal epileptiform discharges as paroxysmal discharges lasting more than 10 s occurring at higher frequency (≥ 2 Hz). If the next ictal event occurs within 10 s after the previous one, we considered the 2 as 1 ictal event. All synchronized bursts that fire in lower frequency (< 2 Hz) or in higher frequency (≥ 2 Hz) with shorter duration (< 10 s) were considered as interictal events. To detect ictal events, signals were preprocessed using a 0.1 to 47 Hz band-pass filter to detrend and to remove electrical noise. We applied a high-pass filter with 10 Hz cutoff to remove the interictal epochs, rectified the signal by quadrating, and applied a low-pass filter with 0.2 Hz cutoff to estimate the signal envelope. We then applied a threshold which only selects the signals that are greater than 3 times the standard deviation above the median value of the whole signal (3 × S.D. + MED). Whole epileptic bursts were detected when the signal amplitude is greater than 6 times the standard deviation of the background noise that was manually selected within the silent period. Of the selected epochs, we considered only the epochs that satisfy the ictal criteria mentioned above. We applied a temporal correction on the start and ending timestamps of each ictal epoch: as long as the ictal burst density is ≥ 2 Hz, the start timestamp is preponed. At the end of the ictal event, the timestamp is postponed as long as the bursts are less than 10 s apart.

To calculate ictal incidence, we categorized slice cultures as either ‘interictal-like’ or ‘mixed ictal- and interictal-like’ activity as described previously [29, 33]. Slices showing only interictal activity were included in ‘interictal-like’ category and slices showing both interictal and ictal activity were included in ‘mixed ictal- and interictal-like’ category.

We also implemented automatic calculation of the power spectrum (PS) for ictal and interictal events in the software using the following equations:

$$ \mathrm{Auto}\ \mathrm{Power}\ \mathrm{Spectrum}=\frac{{\mathrm{FFT}}^{\ast}\left(\mathrm{Signal}\right)\times \mathrm{FFT}\left(\mathrm{Signal}\right)}{n^2} $$

in which *n* is the number of points in the signal and * denotes the complex conjugate. FFT is fast Fourier transform (μV^2^ rms).

### ELISA and Lactate Dehydrogenase Assay

Culture supernatants were collected 2 to 3 times a week and kept at − 80 °C until the assays were performed. All experiments were performed according to the manufacturers’ protocols. In brief, released IL-1β, IL-6, and TNFα were measured from undiluted supernatants using commercially available V-Plex rat ELISA kit (Meso Scale Discovery, Rockville, MD), and electroluminescent signals were detected by MESO QuickPlex (Meso Scale Discovery, Rockville, MD). Lower detection limit of the kit was 6.92 pg/ml for IL-1β, 13.8 pg/ml for IL-6, and 0.72 pg/ml for TNFα. Lactate dehydrogenase (LDH) was measured using LDH assay kit (BioVision, Milpitas, CA). The culture supernatants were diluted two- to threefolds with the assay buffer (BioVision), and colorimetric signals were measured at 450 nm by a microplate reader FlexStation 3 (Molecular Devices, San Jose, CA). LDH concentrations were calculated from the level of LDH activity that converts NAD to NADH. The concentration was expressed as milliunits per milliliter, in which 1 U of LDH generates 1 μM NADH per minute. Goat immunoglobulin G concentration in culture supernatants was measured using the competitive inhibition IgG ELISA kit (Cusabio, College Park, MD). The detection range of the kit was 0.146 to 37.5 μg/ml.

### Double Immunostaining

Hippocampal slices cultured on glass coverslips for 3, 7, 14, and 21 days were fixed by immersion in phosphate buffered saline (PBS) containing 4% paraformaldehyde for 30 min, cryoprotected in PBS containing 30% sucrose, and stored at − 20 °C until staining. TNFα immunostaining was carried out as previously described [17, 44]. Slices on glass coverslips (*n* = 3, 3, 7, and 4 for 3, 7, 14, and 21 DIV, respectively) were incubated in 70% methanol and 2% H_2_O_2_ in Tris–HCl-buffered saline (TBS) at 4 °C for 10 min, followed by 30 min incubation in TBS containing 10% fetal bovine serum (FBS) and 1% Triton X-100. The slices were then incubated overnight at 4 °C in the same buffer containing the primary antibody against TNFα (1:750, Peprotech EC LTD, London, UK). Then, the biotinylated secondary anti-rabbit antibody (1:200, Vector Labs, Burlingame, CA) was applied for 1 h at room temperature. For TNFR1 and TNFR2 staining, slices on glass coverslips (*n* = 5, 6, 10, and 6 for TNFR1; *n* = 7, 7, 10, and 11 for TNFR2 at 3, 7, 14, and 21 DIV, respectively) were incubated in PBS with 0.3% H_2_O_2_ and 0.3% Triton X-100 at 4 °C for 10 min. After three 5 min washing steps with 0.3% Triton X-100 in PBS, slices were incubated in PBS with 10% FBS and 0.3% Triton X-100 at 4 °C for 60 min. The slices were incubated for 72 h at 4 °C in PBS containing 4% FBS, 0.3% Triton X-100, and the biotinylated primary antibody against TNFR1 or TNFR2 (1:30, Hycult Biotech, Uden, Netherlands). Following streptavidin–HRP reaction, the signal was detected by tyramide conjugated to fluorescein using TSA amplification kit (NEN Life Science Products, Boston, MD). No positive staining was observed in the absence of the primary antibodies. Slices were subsequently incubated with the following primary antibodies: mouse anti-CD11b (complement receptor type 3, OX-42, 1:50, Serotec Ltd, Oxford, UK), a marker of microglia-like cells; mouse anti-glial fibrillary acidic protein (GFAP, 1:2500, Chemicon, Burlington, MD), a selective marker of astrocytes; or mouse anti-neuronal specific nuclear protein (NeuN, 1:500, Chemicon, Burlington, MD), a selective neuronal marker. Fluorescence was detected by an anti-mouse secondary antibody conjugated with Alexa546 (Molecular Probes, ThermoFisher Scientific, Gent, Belgium). Slide-mounted slices were examined with an Olympus Fluorview laser scanning confocal microscope (microscope BX61 and confocal system FV500, Olympus, Milan, Italy) using dual excitation of 488 nm (laser Ar) and 546 nm (laser He–Ne green) for fluorescein and Alexa546, respectively. The emission of fluorescent probes was collected on separate detectors. To eliminate the possibility of bleed-through between channels, the sections were scanned in a sequential mode. All images were taken in the CA1 region.

For GFAP and CD11b signal quantification, 3, 7, 14, and 21 DIV hippocampal slices were analyzed as previously described [45]. In each slice, 3 (CA1 and CA3) and 2 (DG hilus) high-power ×60 magnification nonoverlapping images were acquired (microscope BX61 and confocal system FV500) and digitized. GFAP- and CD11b-immunostained areas were analyzed as positive pixels/total assessed pixels using ImageJ software and indicated as staining percentage area. These values were averaged per hippocampal CA1, CA3, and hilus subfield of each slice and used for subsequent statistical analysis.

### Quantitative Polymerase Chain Reaction

At the end of the MEA recordings, cultured slices were collected and kept at − 80 °C until the assay was performed. We used RNeasy Plus Universal Mini kit (Qiagen, Antwerp, Belgium) to obtain total RNA from tissues. After homogenization of the slices by mixing with vortex in QIAzol Lysis Reagent, RNA was purified following the manufacturer’s instructions. RNA concentration was measured with a NanoDrop ND-1000 Spectrophotometer (ThermoFisher Scientific, Gent, Belgium). RNA quality was assessed using the Bio-Rad (Temse, Belgium) Experion Automated Electrophoresis System (RQI > 9). cDNA was then synthesized from 250 ng total RNA using Applied Biosystems High-Capacity cDNA Reverse Transcription Kit (ThermoFisher Scientific, Gent, Belgium) in a total volume of 25 μl following the manufacturer’s protocol. Quantitative PCR reactions (qPCR) were performed using a CFX384 Real-Time System. Four times diluted cDNA (2 μl) was analyzed in duplicate for genes of interest (Suppl. Table 1) expression using inventoried or made to order TaqMan® Gene Expression Assays (ThermoFisher Scientific, Gent, Belgium) and TaqMan® Universal PCR Master Mix (ThermoFisher Scientific, Gent, Belgium) in a final volume of 10 μl according to the manufacturer’s recommendations. Cq values were obtained from Bio-Rad CFX Manager 3.1 software using regression determination mode. Normalized relative expression levels were calculated using qbase+ software [46] (Biogazelle NV, Gent, Belgium). Among 10 potential reference genes tested, PpiB and Hprt1 were identified with the geNormplus [47] module in qbase+ as the most suitable reference genes and were used for normalization.

For principal component analysis (PCA), we used normalized expression values from 20 genes (Suppl. Table 1) at 3 time points (7, 14, and 21 DIV). PCA was performed separately for vehicle- or anti-TNFα antibody-treated slices and compared by graphical plotting of PC1/PC2 distribution.

### Statistics

Statistical differences were assessed by 2-sample *t* test, 1-way or 2-way ANOVA with Tukey’s post hoc analysis, or Fisher’s exact test. *p* < 0.05 was considered significant. Mean ± standard deviation (S.D.) or standard error of mean (S.E.M.) are presented.

## Results

### Development of Epileptiform Activity in OHSCs

We first tested whether epileptiform activity reliably develops in hippocampal slices cultured on MEA surfaces as described in previous studies [33, 34]. Hippocampal slices cultured for 10 days (Fig. 1A) showed mixed ictal- and interictal-like activity across all hippocampal regions (Suppl. Fig. 1). Interictal-like activity was characterized by a single burst with a complex shape containing 1 to several population spikes (Fig. 1B). Ictal-like activity was characterized by a train of spikes that occur at high frequency (2-10 Hz). Extracellular recording using 60 electrodes array enabled us to monitor epileptiform activity in all hippocampal subregions (Fig. 1A) and revealed that the epileptiform activities were highly synchronized in DG, CA3, and CA1 (Suppl. Fig. 1). In order to follow the development of epileptiform activity, we monitored 78 OHSCs at different DIVs and categorized them as either ‘interictal-like’ or ‘mixed ictal- and interictal-like’ activity, as described in the “Methods” section (Fig. 1C). The proportion of slices showing ictal-like activity (incidence) was low (2 out of 12 slices; 16.7%) at early stage (3 DIV) then progressively increased during the following days in culture (23.8%, 50%, and 85.7% at 7, 14, and 21 DIV, respectively). We calculated also the number and duration of ictal activity. There was a clear and significant progression in number of ictal events per slice from 3 to 21 DIV (Fig. 1D). Furthermore, ictal firing accounted for only 1.9 ± 1.7% of the entire recording time at 3 DIV, gradually evolved, and reached to 33.5 ± 22.3% at 21 DIV (Fig. 1E). Duration of individual ictal event also progressively increased (Fig. 1F). We performed power spectrum analysis in 0 to 30 Hz frequency range and found that the group of slices at the later stages (14-21 DIV) showed significantly higher power than the group at 3 to 7 DIV in the frequency range between 5 and 30 Hz (Fig. 1G). These results indicate a progression of epileptiform activity in OHSCs which paralleled the age of the culture.

### Inflammation in OHSCs

**Fig. 2:**
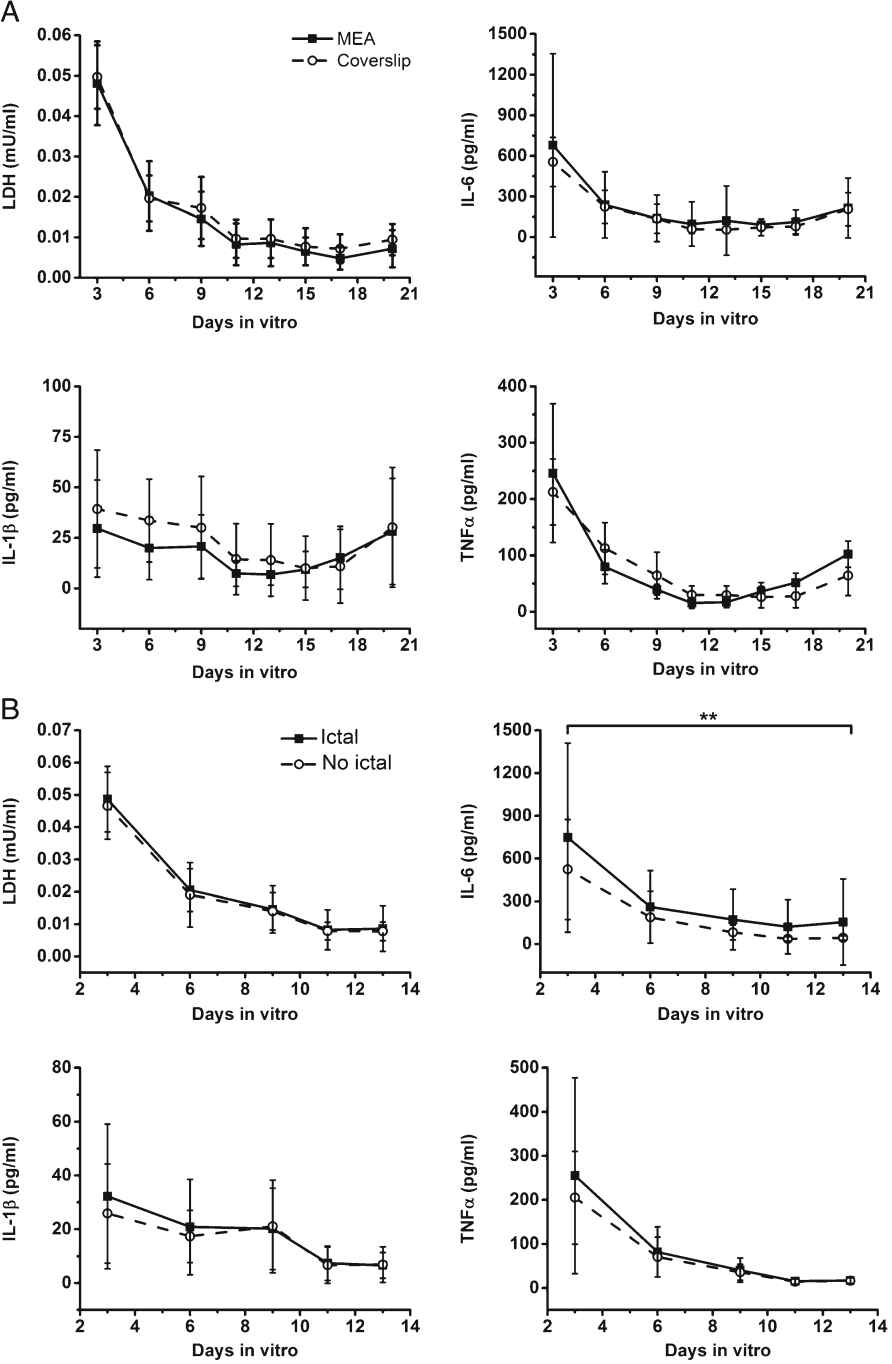
Time course of pro-inflammatory cytokines and LDH release in OHSCs. (A) The inflammatory cytokines IL-1β, TNFα, and IL-6 and a cell death marker LDH were measured in medium collected at 3, 6, 9, 11, 13, 15, 17, and 20 DIV. Levels were compared between slices cultured on MEA (*n* = 15-81) and on coverslips (*n* = 8-33). (B) Comparison of the cytokines and LDH release profile between ictal and interictal (no-ictal) groups from 3 to 14 DIV. For IL-6: **p < 0.01 overall by 2-way ANOVA. All data are presented as mean ± S.D.

**Fig. 3:**
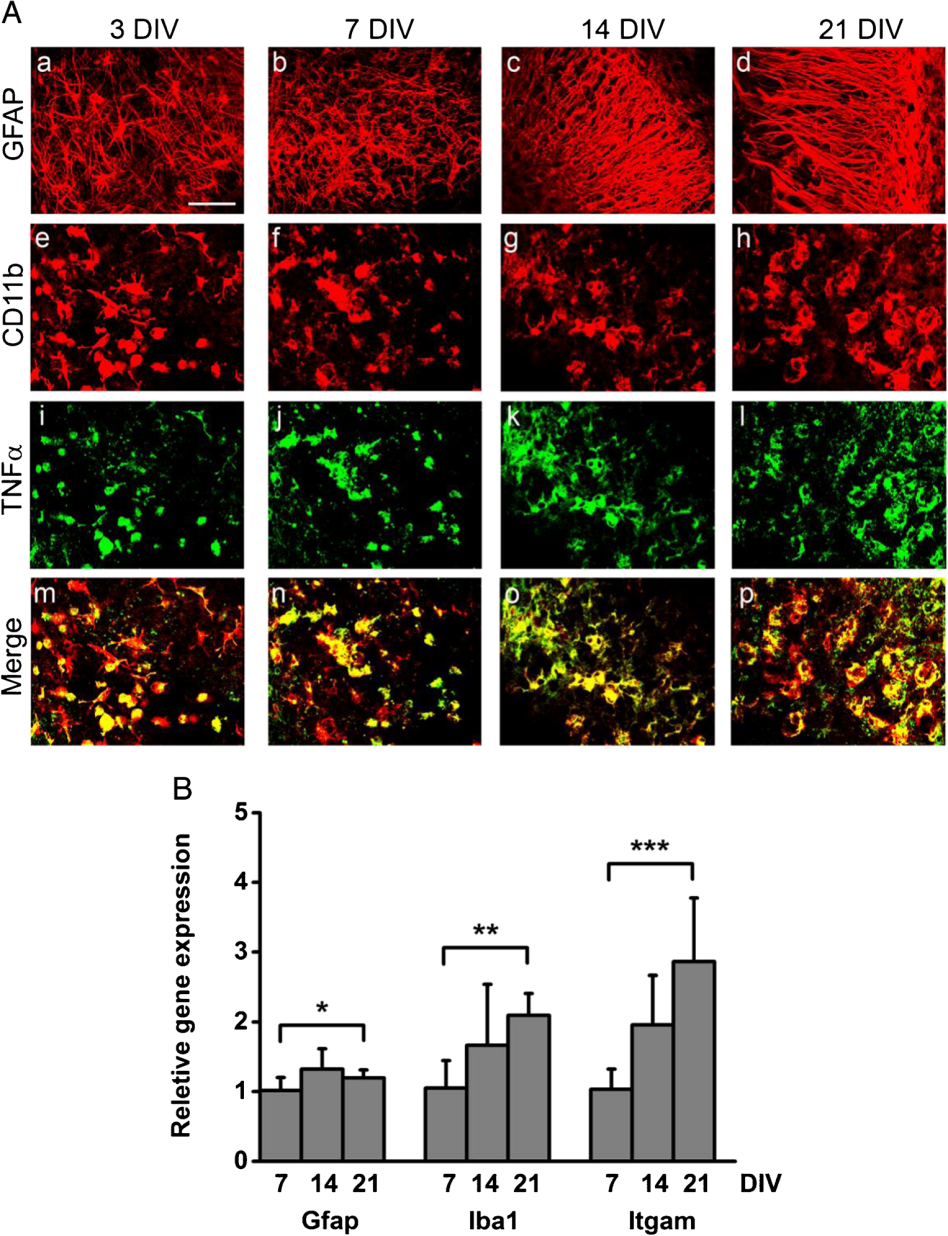
Time course of microglia and astrocytes activation. (A) Double immunostaining for astrocytes, microglia, and TNFα in OHSCs. Panels a–d depict representative images of GFAP-positive astrocytes exhibiting hypertrophic cell body and long processes at 3 (a) and 7 DIV (b). GFAP-positive astrocytes show a fibrous morphology with long and thick processes at 14 (c) and 21 DIV (d). Panels e–h depict representative images showing immunopositive microglia-like cells at 3 (e), 7 (f), 14 (g), and 21 (h) DIV. Expression of TNFα (green) and double staining with CD11b (red) at different stages are shown in panels i–l and m–p, respectively. All images were taken in the CA1 region. Scale bar, 50.0 μm. (B) Gene expression profiles of astrocytic and microglial markers. Expression of *gfap*, *iba1*, and *itgam* (cd11b) was quantified by RT-qPCR at 7, 14, and 21 DIV. *Gfap*: overall *p < 0.05, pairwise comparison *p* < 0.01 for 7 *versus* 14 DIV; *Iba1*: overall **p < 0.01, pairwise comparison *p* = 0.001 for 7 *versus* 21 DIV; *Itgam*: overall ***p < 0.001, pairwise comparison *p* < 0.05 for 7 *versus* 14, *p* < 0.001 for 7 *versus* 21, and *p* < 0.05 for 14 *versus* 21 DIV. *n* = 9 to 10 slices/DIV. Statistical differences were assessed by 1-way ANOVA followed by Tukey’s post hoc test. Data are presented as mean ± S.D.

To examine the cytokine release profile during the development of epileptiform activity, we measured the 3 prototypical pro-inflammatory cytokines—IL-1β, IL-6, and TNFα—in culture supernatants that were collected from 3 to 20 DIV (Fig. 2). LDH release was also evaluated in the same medium to assess the extent of cell death in OHSCs. The 3 cytokines showed very similar release profiles over the culture period, although the peak level of IL-6 was higher (678.3 pg/ml) than TNFα (246.0 pg/ml) and IL-1β (29.6 pg/ml) (Fig. 2A). The most elevated level of cytokine release happened during the period of 0 to 3 DIV with high LDH secretion suggesting that this release is probably caused by the tissue damage during slice preparation. After 3 DIV, the cytokine release decreased until a significant surge was observed at 20 DIV (Suppl. Fig. 2), a time at which the LDH release was low. Notably, IL-1β level was below the detection limit of our assay (6.92 pg/ml) in several samples analyzed between 3 and 20 DIV. We also examined whether the slices undergoing ictal events showed a distinct release pattern compared with slices with interictal (nonictal) activity from 3 to 14 DIV (Fig. 2B). We could not compare this pattern after 14 DIV, because more than 80% of slices showed ictal activity from this time point; therefore, the sample size in the interictal group was too small to perform statistical analysis. No difference was observed in IL-1β, TNFα, and LDH release, whereas IL-6 level was significantly lower in the nonictal than in the ictal group. Because we later performed immunostaining in slices cultured on coverslips, we compared the cytokines and LDH release measured in slices cultured on MEAs *versus* glass coverslips (Fig. 2A). Comparable profiles of release were observed in cultures on the two surfaces; therefore, we performed immunocytochemical staining to study the time course of inflammatory markers in OHSCs cultured on glass coverslips at 3, 7, 14, and 21 DIV. GFAP, an astrocytic marker, was present in hypertrophic astrocytes with long and thick processes at both 3 and 7 DIV (Fig. 3A, panels a, b). At 14 and 21 DIV, the majority of GFAP-positive cells had enlarged cell bodies and long processes (c, d). We also observed that the astrocytic processes at the later stages (14 and 21 DIV) tended to be aligned together, which was not the case at the earlier stages (3 and 7 DIV). Alignment of astrocytes is thought to help neuronal regeneration after injury [48]. CD11b (complement receptor type 3), a microglial marker, was also strongly expressed in cells with large cell bodies and short processes; these cells often exhibited phagocytic features with a rounded amoeboid phenotype (Fig. 3A, panels e–h). The morphologically active microglia was observed at all stages. GFAP and CD11b signal quantification revealed a progressive increase in GFAP- and CD11b-stained area with the highest expression levels at 14 and 21 DIV (Suppl. Fig. 3). No subregional difference in glial activation was observed in CA1, CA3, and DG hilus. RT-qPCR data also showed significant increases of *gfap*, *iba1* (ionized calcium-binding adapter molecule 1), and *itgam* (integrin alpha M, Cd11b) expression at later stages (Fig. 3B), thus supporting the time-dependent activation of astrocytes and microglia in OHSCs.

**Fig. 4:**
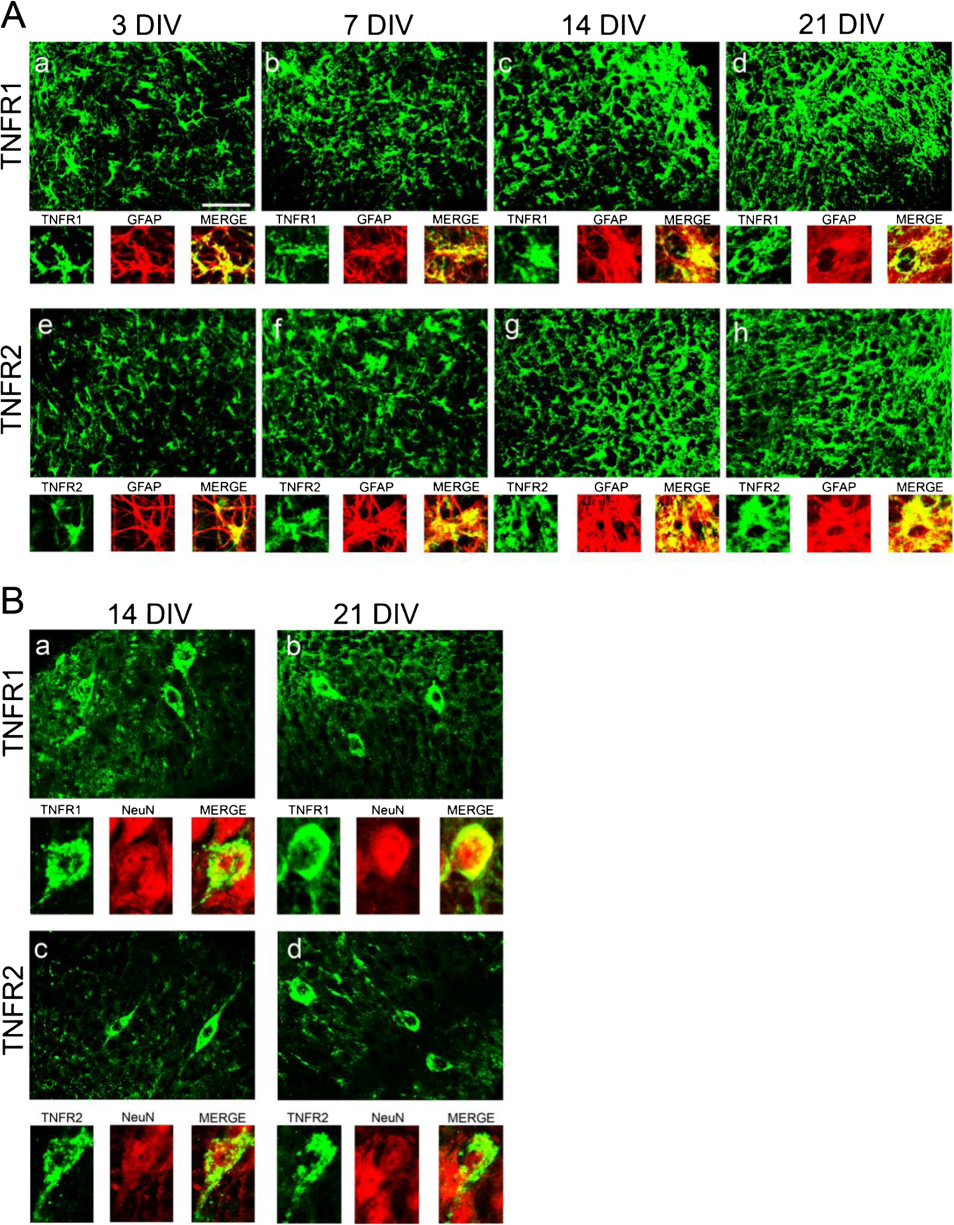
Immunofluorescence staining for astrocytic and neuronal localization of TNFR1 and TNFR2 in OHSCs. (A) Representative images depict immunofluorescence signals of TNFR1 (panels a–d) and TNFR2 (e–h) at 3, 7, 14, and 21 DIV. High magnification images show TNFR1 and TNFR2 (green) and GFAP-positive astrocytes (red) and their co-localization (merge, yellow) at all stages. (B) The images show TNFR1 (a, b) and TNFR2 (c, d) at 14 and 21 DIV in CA1 pyramidal layer. High magnification images depict co-localization (merge, yellow) of TNFR1 or TNFR2 (green) with NeuN-positive neurons (red) at 14 and 21 DIV. Scale bar, 50.0 μm; high magnification panels, 25.0 μm. All images were taken in the CA1 region

Because our study was focused on the TNFα pathway, we immunolabeled OHSCs with antibodies against TNFα and its two receptors, TNFR1 and TNFR2. Figure 3A (panels i–l) shows representative images of TNFα immunoreactivity in OHSCs. This cytokine was strongly expressed throughout the whole slice in cells with glia morphology at all stages. Double-labeling experiments revealed that TNFα is predominantly expressed in CD11b-positive microglia (Fig. 3A, panels m–p). TNFR1 and TNFR2 immunoreactivity was observed in glia-like cells and double staining revealed that both receptors were mainly expressed in GFAP-positive astrocytes (Fig. 4A, high magnification panels). At 14 and 21 DIV, TNFR1 (Fig. 4B, panels a, b) and TNFR2 (c, d) were additionally expressed by NeuN-positive pyramidal neurons (high magnification panels).

### Phenytoin Does Not Affect Epileptogenesis

**Fig. 5:**
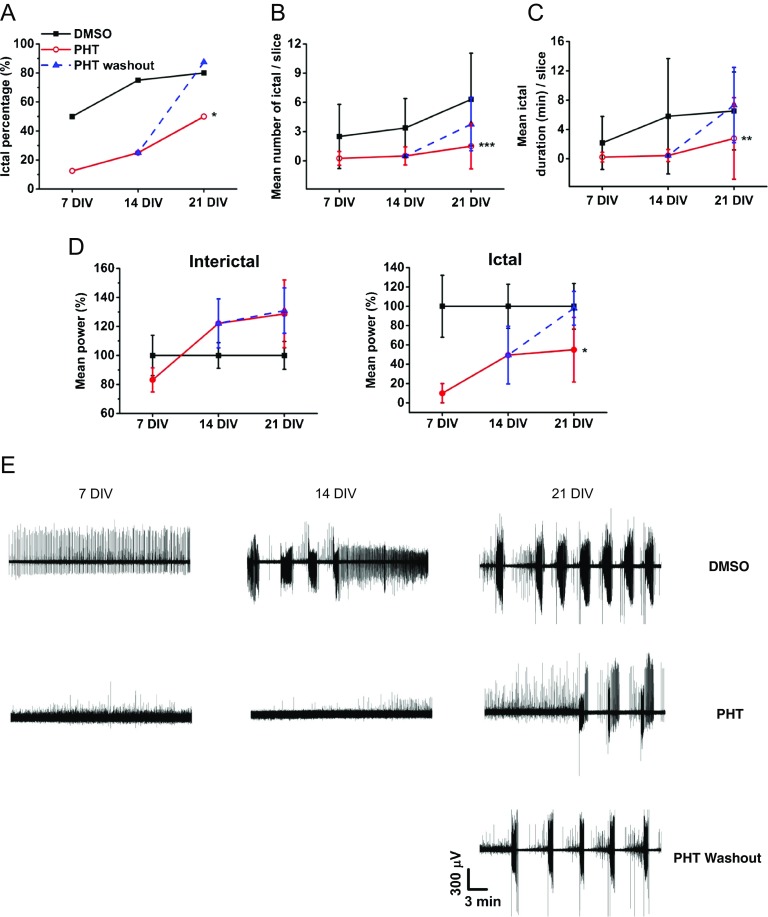
Effect of phenytoin (PHT) on ictal development in OHSCs. (A) Incidence of ictal events (%) was calculated by ictal slice/total number of recorded slices in 0.1% DMSO (vehicle), PHT (present from 0 to 21 DIV) and PHT washout (present from 0 to 14 DIV) groups. Values and sample size are presented in Table 1. Fisher’s exact test *p = 0.01 DMSO *versus* PHT for whole period. (B, C) Mean number and duration of ictal events were analyzed and compared among groups. (B) Two-way ANOVA overall ***p < 0.001 PHT *versus* DMSO, pairwise comparison *p* < 0.05 for 7 *versus* 21 DIV. (C) Two-way ANOVA overall **p < 0.01 PHT *versus* DMSO, no significant differences on DIVs. Data are presented as mean ± S.D. (D) Power of interictal and ictal events were analyzed to quantify epileptic signal intensity. Significant reduction in ictal power was observed in the PHT group compared to the DMSO group. Overall *p = 0.01 PHT *versus* DMSO. Two-way ANOVA followed by Tukey’s post hoc test. Data are presented as mean ± S.E.M. (E) Representative traces of epileptiform activity in DMSO, PHT, and PHT washout groups at 7, 14, and 21 DIV

Phenytoin (PHT) is a first-generation AED widely used in the clinic for treating tonic–clonic and partial seizures [5]. We tested whether PHT affects the development of epileptiform activity in OHSCs by analyzing ictal and interictal events at 7, 14, and 21 DIV. We divided PHT-treated slices into two groups: one group was cultured in the presence of 50 μM PHT from 0 to 21 DIV, whereas in the other group, PHT was washed out at 14 DIV. PHT efficiently reduced the incidence of ictal events at all DIVs compared to 0.1% DMSO-exposed slices used as the control group (Table 1 and Fig. 5A). However, washout of PHT at 14 DIV resulted in a rapid increase in incidence of ictal events at 21 DIV indicating that PHT did not prevent ictal progression in OHSCs. Mean number, duration as well as power of ictal events were significantly decreased by PHT during treatment, but these effects elapsed after drug washout (Fig. 5B–E). The power of interictal events was not different among the groups during the entire period (Fig. 5D). To note, ictal development in OHSCs chronically treated with 0.1% DMSO seemed faster and more severe when compared with the untreated slices grown in the culture medium only (DMSO in Table 1 and vehicle in Table 2).

**Table 1:** Incidence of ictal events in the DMSO, PHT, and PHT washout groups

DIV	Incidence of ictal %		
	DMSO	PHT*	PHT (washout)
7	50 (4/8)	12.5 (1/8)	
14	75 (6/8)	25 (2/8)	
21	80 (8/10)	50 (4/8)	87.5 (7/8)

Incidence of ictal (%) was calculated by ictal slice/total number of recorded slices (in brackets). Fisher’s exact test *p = 0.01 for DMSO *versus* PHT for the whole period

**Table 2:** Incidence of ictal events in the vehicle and anti-TNFα group

DIV	Incidence of ictal %	
	Vehicle	Anti-TNFα**
7	41.7 (5/12)	0.8 (1/12)
14	43.8 (7/16)	14.3 (2/14)
21 (washout)	78.6 (11/14)	46.2 (6/13)

Incidence of ictal (%) was calculated by ictal slice/total number of recorded slices (in brackets). Fisher’s exact test **p < 0.01 for the vehicle *versus* anti-TNFα for the whole period

**Fig. 6:**
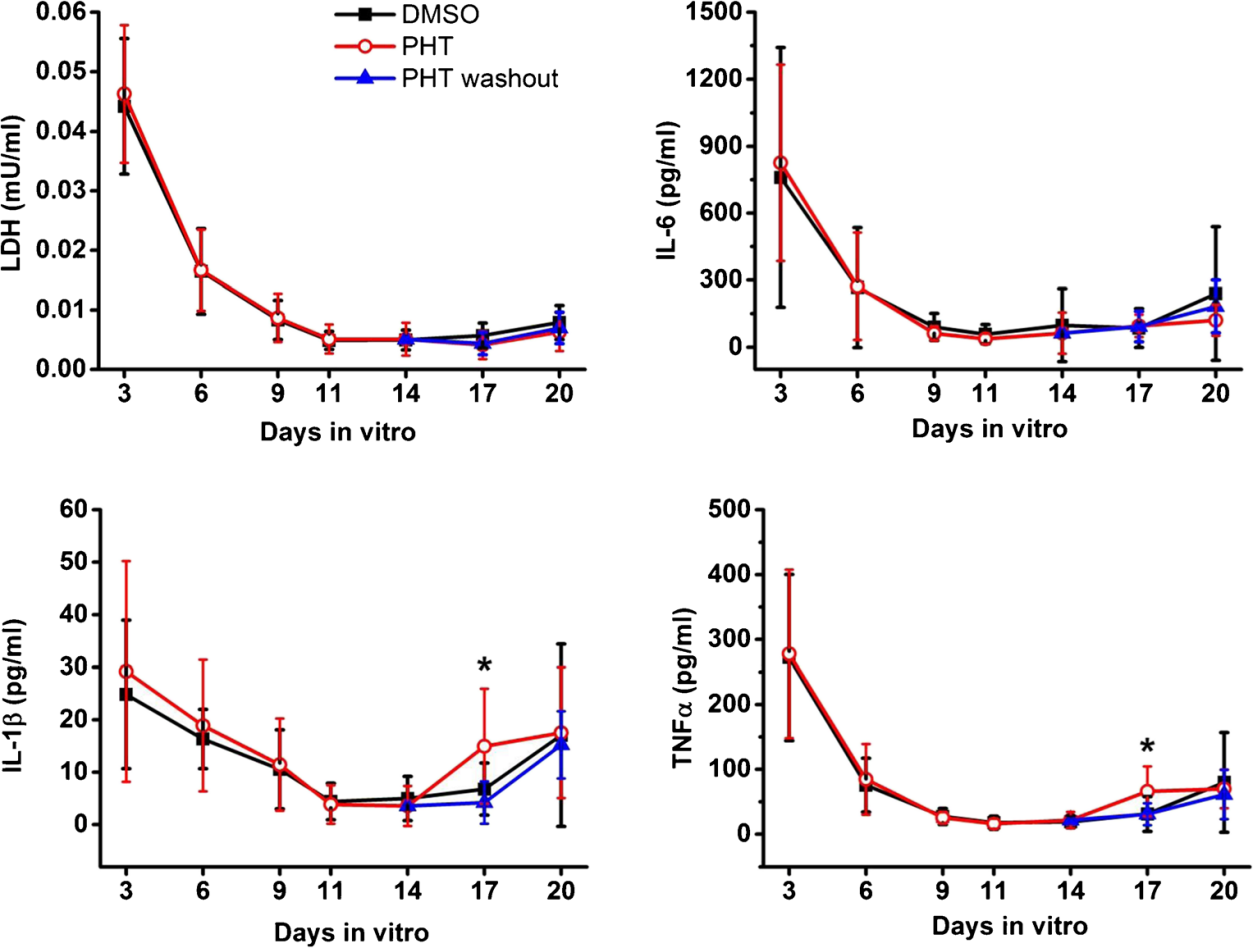
Effect of phenytoin (PHT) on cytokine and LDH release in OHSCs. Secretion level of the inflammatory cytokines IL-1β, TNFα, and IL-6 and cell death marker LDH was quantified at 3, 6, 9, 11, 14, 17, and 20 DIV and compared among DMSO, PHT, and PHT washout groups. Two-sample *t* test IL-1β *p < 0.05 at 17 DIV for DMSO *versus* PHT and PHT *versus* PHT washout; TNFα *p < 0.05 at 17 DIV for DMSO *versus* PHT and PHT *versus* PHT washout. *n* = 11 to 27 for DMSO, 8 to 32 for PHT, and 8 slices for PHT washout. Data are presented as mean ± S.D.

PHT did not alter the overall profiles of the cytokines and LDH release (Fig. 6). A transient increase in IL-1β and TNFα release was seen at 17 DIV in slices continuously exposed to PHT (IL-1β 14.9 ± 11.0 pg/ml in the PHT group and 6.8 ± 5.0 pg/ml in the DMSO group; TNFα 66.0 ± 38.6 pg/ml in the PHT group and 31.1 ± 26.9 pg/ml in the DMSO group, mean ± S.D.). Interestingly, this increase elapsed when PHT was washed out (IL-1β 4.2 ± 4.0 pg/ml and TNFα 30.8 ± 16.9 pg/ml, mean ± S.D.) indicating that it was driven by the presence of PHT. Our data therefore support the evidence that PHT acutely reduces neuronal network hyperexcitability but does not prevent or modify the epileptogenic process in this *in vitro* model in accordance with clinical evidence in TBI-exposed patients developing epilepsy [29, 49, 50].

### Anti-TNFα Antibody Delays the Development of Ictal-Like Activity

**Fig. 7:**
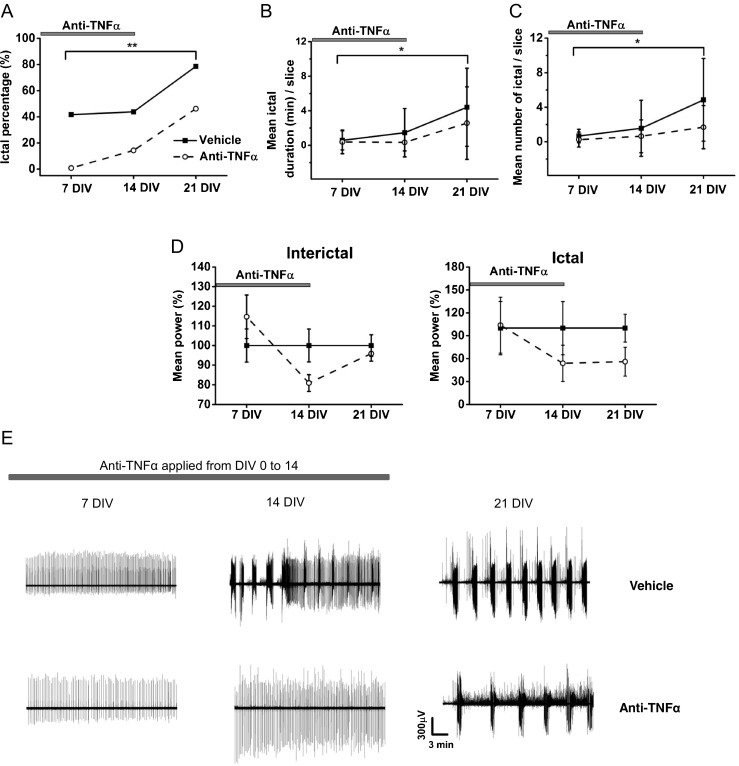
Effect of anti-TNFα polyclonal antibody on the development of ictal activity in OHSCs. (A) Incidence of slices developing ictal activity (%) in anti-TNFα and vehicle-treated slices was calculated by ictal slice/total number of recorded slices. Values and sample size are presented in Table 2. Fisher’s exact test **p < 0.01 vehicle *versus* anti-TNFα antibody for the whole incubation period including washout period. (B, C) Mean number and duration of ictal events were analyzed and compared between anti-TNFα antibody and vehicle groups. (B) Two-way ANOVA overall *p < 0.05 vehicle *versus* anti-TNFα, pairwise comparison *p* = 0.001 for 7 *versus* 21 DIV, *p* = 0.01 for 14 *versus* 21 DIV. (C) Two-way ANOVA overall *p < 0.05 vehicle *versus* anti-TNFα, pairwise comparison *p* = 0.001 for 7 *versus* 21 DIV, *p* < 0.01 for 14 *versus* 21 DIV. Data are presented as mean ± S.D. (D) Power of interictal and ictal events were analyzed to quantify epileptic signal intensity. No significant difference was observed between the 2 groups. Data are presented as mean ± S.E.M. (E) Representative traces of epileptiform activity of vehicle- and anti-TNFα-treated slices at 7, 14, and 21 DIV

Our results demonstrate a high level of inflammation during epileptogenesis in OHSCs, represented by persistent microglia and astrocyte activation and especially high levels of TNFα and IL-6 release. To address the question whether blocking TNFα actions modulates the development of ictal-like activity, we used an anti-TNFα polyclonal antibody (10 μg/ml) to neutralize soluble TNFα. The antibody was continuously present in the culture medium from 0 to 14 DIV and washed out at 14 until 21 DIV. The development of ictal event was evaluated at 7, 14, and 21 DIV. The antibody significantly decreased the incidence of ictal-like activity at all stages, notably also after washout (Table 2 and Fig. 7A, E). Occurrence of ictal events at 7, 14, and 21 DIV was much lower in the anti-TNFα group (0.8%, 14.3%, and 46.2%) than in the vehicle group (41.7%, 43.8%, and 78.6%). Although the age-dependent ictal progression was still observed in the group treated with anti-TNFα, the incidence of ictal activity remained significantly lower after TNFα antibody washout than in the vehicle group. We also found a significant reduction in all parameters of ictal firing between the treated *versus* vehicle groups (Fig. 7B, C). The mean number and duration of ictal events were significantly lower indicating that the antibody application efficiently reduced ictal-like activity. Taken together and in contrast to the results obtained with PHT, these results strongly suggest that the effect of anti-TNFα is not an anti-seizure but anti-epileptogenic because the attenuation of epileptic markers—mainly ictal discharges—is maintained after removal of the anti-TNFα antibody. The mean power of ictal and synchronized interictal-like activity showed a trend to decrease from 14 DIV in the treated slices; however, the difference was not significant (Fig. 7D).

Because we observed a substantial increase of IL-6 release during epileptogenesis and significantly higher level in ictal slices than in nonictal slices (Fig. 2A, B), we also examined the effect of blocking IL-6 on the development of ictal-like activity. Anti-IL-6 polyclonal antibody (10 μg/ml) was applied to the culture medium from 0 to 14 DIV and washed out at 14 DIV. Unlike the effect observed with the anti-TNFα antibody, anti-IL-6 antibody was ineffective in delaying the development of ictal-like activity in OHSCs (Suppl. Fig. 4). Incidence, number, and duration of ictal events in the anti-IL6 group were very similar to those in the vehicle group.

**Fig. 8:**
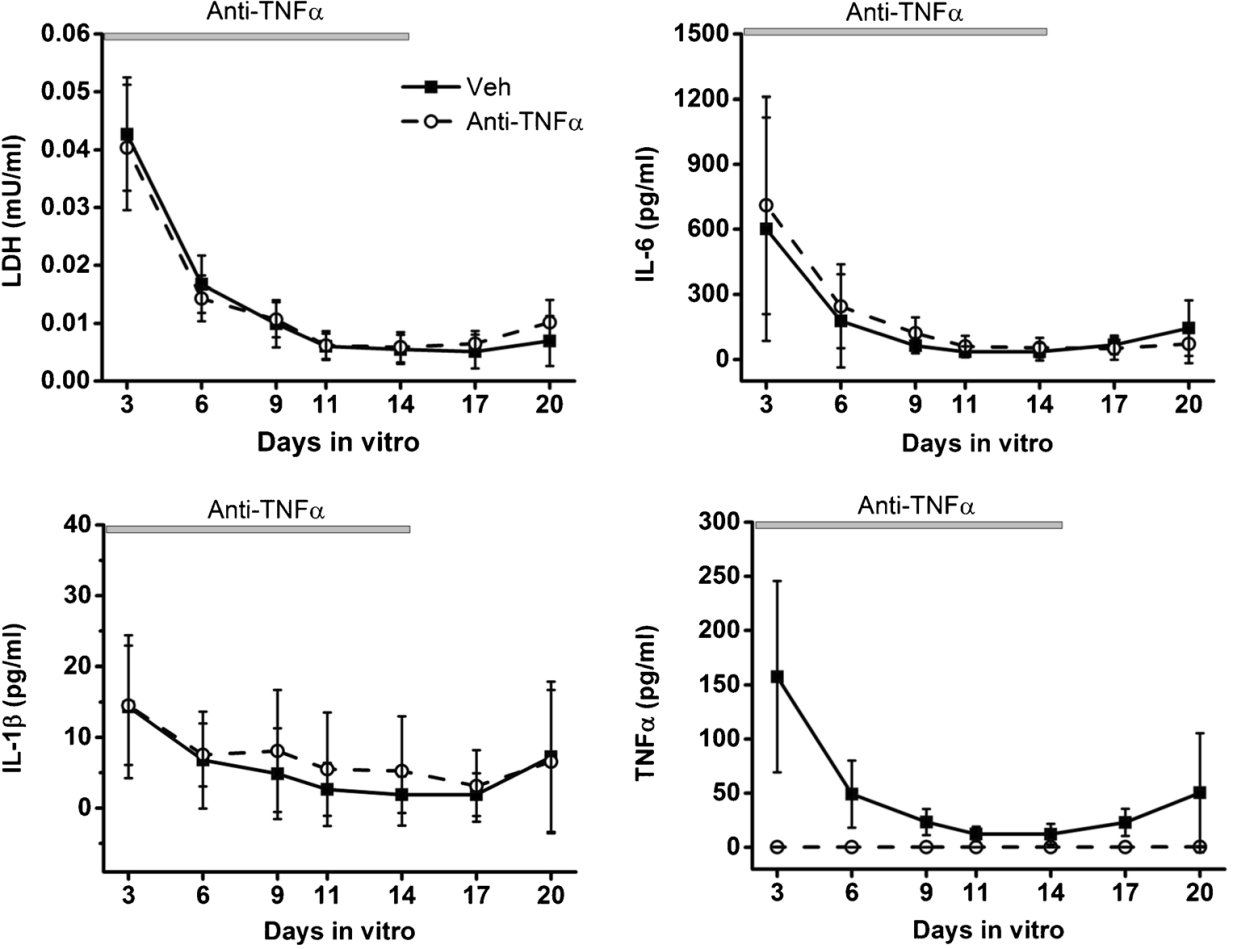
Effect of anti-TNFα polyclonal antibody on cytokine and LDH release in OHSCs. Supernatant level of inflammatory cytokines IL-1β, TNFα, and IL-6 and LDH was quantified at 3, 6, 9, 11, 14, 17, and 20 DIV and compared between anti-TNFα antibody and vehicle groups. *n* = 10 to 29 for anti-TNFα and 14 to 33 slices for vehicle. Data are presented as mean ± S.D.

We also measured the pro-inflammatory cytokine release from anti–TNFα-antibody-treated slices. The overall profiles of IL-1β, IL-6, and LDH release did not significantly differ between the anti-TNFα antibody and the vehicle groups (Fig. 8). Importantly, soluble TNFα level dropped below the detection limit in the treatment group confirming efficient neutralization of TNFα by the polyclonal antibody. Notably, TNFα level remained at a low level (0.5 pg/ml) after the antibody was washed out suggesting that blocking soluble TNFα for 14 days has resulted in lasting inhibition of its own release. We checked if any residual antibody was detected in culture supernatants after washout using the ELISA that detects goat immunoglobulin G (IgG) in supernatants collected before (14 DIV) and after washout (16 and 20 DIV). Goat IgG titration was above the neutralizing dose 50 (ND50) at 14 DIV (1.00 ± 0.38 μg/ml) then decreased to 0.13 ± 0.10 and 0.008 ± 0.01 μg/ml at 16 and 20 DIV, respectively (Suppl. Fig. 5). According to the manufacturer’s information, more than 90% of free recombinant TNFα remains unbound by the antibody concentration of 0.13 ± 0.03 μg/ml. Therefore, the possibility that residual antibody neutralizes soluble TNFα after the washout is very unlikely.

To understand the mechanisms underlying delayed ictal development after incubation with the anti-TNFα antibody, vehicle or anti–TNFα-treated slices were collected after MEA recordings to perform gene expression studies using RT-qPCR. We selected 20 genes that are involved in innate inflammation and in TNFR1 and R2 signaling pathways (Suppl. Table 1) [51, 52]. To understand how gene expression profiles change in function of time and treatment, PCA was performed independently on vehicle- and anti–TNFα-treated samples at the 3 time points, and samples were plotted on PC1/PC2 scatter (Suppl. Fig. 6). Vehicle-treated samples showed homogeneous clustering at each time point and clear separation between 7 and 14/21 DIV for the 20 genes analyzed. This clustering, however, was absent in anti–TNFα-treated samples. After analysis of the variables that most significantly contribute to PC1, we identified 6 genes (*fadd, map3k1, adam17, caspase-8, tnrf1, and tnfr2*) whose expression levels increased in the vehicle group over time. The time-dependent increase in these genes was absent in the anti-TNFα group (Suppl. Table 2). Overall, the data indicate that the neutralization of TNFα, in contrast to IL-6 neutralization and PHT, delays the development of epileptifom activity in OHSCs and reduces the number and duration of ictal events. These effects are possibly associated with specific and time-dependent changes in TNF-related signaling pathways.

## Discussion

The main findings of this study indicate that both age-dependent ictal progression and inflammation driven by activated microglia and astrocytes are present in the OHSCs. Furthermore, we provide evidence that the inhibition of TNFα release delays ictal development in this model. Microglia and astrocytes displayed “reactive” morphology throughout the culture period (3 to 21 DIV) which is comparable to the previous study describing chronic inflammation in OHSCs [53]. The release of pro-inflammatory cytokines IL-1β, TNFα, and IL-6 peaked at 3 DIV concomitantly with substantial cell death (LDH release) and declined to minimal levels around 10 DIV. A second wave of cytokine release was observed around 20 DIV when LDH secretion was very low, thus suggesting it was not caused by cell death. Although intrinsic inflammation remained elevated, epileptiform activity spontaneously evolved in OHSCs. Interestingly, Berdichevsky et al. [29] observed 2 phases of LDH increase in OHSCs and found that the first LDH increase between DIV 0 and 3 reflects post-traumatic cell death due to slicing but that the mild secondary increase after 10 DIV is due to the severe ictal progression. Importantly, blocking this secondary cell death did not affect the epileptogenesis confirming that the activity-dependent cell death is not a driving factor of the development of epilepsy in this model.

The OHSC–MEA platform we used offers an easy and simultaneous access to various experimental readouts which is difficult to achieve using *in vivo* models. For example, the development of epileptiform activity can be directly monitored from OHSCs grown on noninvasive MEA electrodes, whereas culture supernatants are a valuable source for measuring dynamic changes in released molecules. Additionally, slices can be used to investigate the expression of gene or protein of interest. Finally, this *in vitro* model allows pharmacological studies that are not suitable for systemic injection in animal models of epileptogenesis due to poor blood–brain barrier permeability. Integration of these data allows understanding of biological processes developing after traumatic injury of brain tissue. Taking the advantage of this platform, we report a detailed time course of epileptogenesis and inflammation after traumatic injury in OHSCs.

The OHSC model reliably mimics cellular features of epileptogenesis, e.g., progressive development of ictal- and interictal-like bursts [29, 33], mossy fiber sprouting in the dentate gyrus [54, 55], and synaptic reorganization of the hippocampal trisynaptic circuit [37, 56]. Moreover, OHSCs maintain a high degree of the original structural organization with comparable developmental and electrophysiological properties to *in vivo* settings [57, 58]. Non-neuronal compartments such as oligodendrocytes and blood vessels are preserved in OHSCs [59–61]. Morphology and distribution of microglia and astrocytes are also similar to the *in vivo* situation after trauma. Thus, massively activated microglia are observed in an early phase following tissue slicing [53, 62, 63], resembling the excessive glial activation induced by trauma *in vivo*. Meanwhile, there are several limitations in this preparation. Hippocampal slices are isolated from the afferent and efferent circuitry, particularly the entorhinal cortex, leading to the collateral sprouting [55]. Although blood vessels are preserved to a certain extent, the absence of a BBB results in lack of neurovascular interactions that are important for supplying neurotrophic factors, systemic inflammatory molecules, and many others [64].

Because of the observed increase in the release of pro-inflammatory cytokines in OHSCs, we examined whether suppressing TNFα action could impact on the development of epileptiform activity. Neutralization of soluble TNFα released in the course of traumatic injury significantly reduced ictal discharges and, importantly, the effect lasted for at least 1 week after antibody washout implying a potential anti-epileptogenic effect in this model.

The polyclonal TNFα antibody used in this study has higher specificity to soluble (sTNFα) over transmembrane TNFα (tmTNFα) [65]. Our ELISA data demonstrate total neutralization of sTNFα in the culture supernatants indicating that the antibody effects in OHSCs depend on efficient inactivation of sTNFα. We did not observe significant effects of the IL-6 antibody on the development of epileptiform activity which also excludes the possibility of nonspecific effects mediated by the presence of IgG in the culture medium.

The relevance of the potential anti-epileptogenic effect of TNFα neutralization is supported by the lack of effect of PHT and the anti-IL-6 antibody on the development of ictal-like activity. PHT has been extensively tested for an anti-epileptogenic effect in TBI patients [49, 50, 66, 67]. PHT was very efficacious in suppressing early seizures but did not prevent the development of epilepsy in those patients. The anti-convulsant action of PHT failed to prevent epileptogenesis in OHSCs that is consistent with our results [29]. Interestingly, we observed transient increase of IL-1β and TNFα release in PHT-treated slices, which was reversible when PHT was washed out. This phenomenon might be due to PHT’s ability to increase platelet-derived growth factor B and pro-inflammatory cytokine production via activation of macrophages and monocytes resulting in gingival overgrowth in humans, which is one of its known side effects [68, 69]. On the other hand, we did not see any beneficial or detrimental effects of blocking IL-6 signaling in the development of epileptiform activity in OHSCs. IL-6 is mainly considered as a pro-inflammatory cytokine, but it also has anti-inflammatory and regenerative properties that are important for recovery after injury [70]. In particular, increased IL-6 release after injury promoted functional recovery including tissue regeneration and neuronal network repair in OHSCs [71, 72]. Elevated IL-6 level in serum or cerebrospinal fluid has been widely observed in patients as well as in animal models with different types of epilepsy [73–75]. However, there have been conflicting reports regarding the role of IL-6 in seizures and epilepsy. Intervention of IL-6 pathway resulted in increased or decreased seizure severity in different rodent models [76–79]. Because many events including injury, response to injury, neuronal regeneration, and epileptogenesis are ongoing in the OHSC model, it is difficult to understand the effect of blocking the multifunctional properties of IL-6 on the development of epilepsy.

The role of TNFα in the CNS is complex and not completely understood so far. In healthy brain, TNFα is crucially involved in host defense mechanisms and other physiological functions such as neural and glial transmission, synaptic plasticity, and astrocyte-mediated synaptic strength [80–82]. TNFα may also exert protective effects against ischemia, multiple sclerosis, excitotoxic damage, and oxidative stress. In pathological conditions, however, excessive TNFα production by activated microglia and astrocytes promotes acute injury and sustains inflammation leading to neuronal hyperexcitability and cell death [83–86]. TNFR1 is thought to play a dominant role in these deleterious effects when compared with TNFR2 which was shown to mediate neuroprotective functions [51, 52, 87].

Although the role of TNFα in epileptogenesis has not been explored, preclinical and clinical evidence suggest its critical involvement in seizure mechanisms. Differential roles of the two TNFα receptors have been proposed in epilepsy. In particular, selective activation of TNFR1 increased seizure susceptibility, whereas activation of TNFR2 decreased seizures in kainic acid-treated animals and in the kindling model [23, 24]. TNFR1 components including TNFR-associated protein with death domain (TRADD), Fas-associated protein with death domain (FADD), and caspase-8 were found to be upregulated in brain samples from TLE patients with intractable epilepsy [88] and contribute to experimental seizures [22, 23]. Interestingly, Savin et al. [89] demonstrated in a computational neuron–glia interaction model that TNFα overexpression by glial cells influences synaptic scaling that leads to increased seizure susceptibility. Their model also predicted that TNFα diffusion from lesion sites may be responsible for epileptogenesis. More recently, a study reported that anti-TNFα therapy using adalimumab significantly decreased seizure frequency in Rasmussen’s encephalitis patients [90]. Collectively, these data provide strong evidence in implication of TNFα on ictal and epileptogenic processes.

To understand the molecular mechanisms underlying the anti-TNFα antibody effect on epileptogenesis in OHSCs, we analyzed mRNA expression profiles related to TNFα-associated pathways. We did not detect significant differences in mRNA levels of the individual genes of interest between the vehicle and the anti-TNFα antibody groups, but we found that the anti-TNFα antibody altered their overall expression patterns. The expression of TNFα-related genes in the vehicle group increased during epileptogenesis as demonstrated by the homogenous clustering at each time point (7, 14, and 21 DIV) in PCA. Anti-TNFα treatment disrupted this clustering pattern during the culture period. In particular, the expression levels of *fadd, map3k1, adam17, caspase-8, tnfr1*, and *tnfr2* increased from 7 to 21 DIV in the vehicle group, while their levels did not increase over time in the anti-TNFα group. This result implies gene alterations in the TNFR1 pathway [51, 52]. Our data prompt future investigations using genome-wide approaches to investigate broader gene expression changes induced by the TNFα antibody. Moreover, RT-qPCR performed on the whole hippocampus should be complemented using single-cell RNA sequencing techniques. Correlation of mRNA expression changes with target protein levels, post-translational modifications, and cellular localization will also help dissect out the critical molecular pathways involved in TNFα effects.

In our studies, we only applied anti-TNFα treatment from DIV 0 to examine the impact of neutralizing TNFα from the initiation of traumatic injury and if this time of intervention affects epileptogenesis. We believe that this experimental design has potential clinical interest because acute inflammation is often involved in the early phase of TBI, and in some patients, seizures occur very early (24 h up to 7 days) [91, 92]. However, it will be also interesting to investigate the effect of neutralizing soluble TNFα at different time points during epileptogenesis. Although the OHSC model shares many mechanistic and physiological factors with human TBI [9, 92], the extent and severity of the primary injury in OHSCs may impair the possibility to modulate inflammation during epileptogenesis [29, 53]. It has been recently reported that silencing immune systems was ineffective in epileptogenesis [93] because depletion of either microglia or T lymphocytes did not prevent the development of ictal activity in the same post-traumatic epileptogenesis model. The authors suggested a need of a less severe model to reveal modulatory effects of anti-inflammatory molecules. Furthermore, interpretation of the outcome upon complete depletion of the immune system can be complex because not only pathological but also physiological functions of many inflammatory mediators are blocked. We believe that our intervention specifically interfering TNFα does represent a harmful arm of the innate immunity contributing to hyperexcitability. Nevertheless, the OHSC model can still give valuable information on discovering new drug targets and pathways in TBI-induced epilepsy. One good example is the high-throughput *in vitro* OHSC model recently developed by Staley’s group [32]. They screened over 400 compounds and found that celecoxib, a selective cyclooxygenase-2 inhibitor, was most effective at inhibiting ictal activity and ictal cell death in OHSCs. Celecoxib also showed potential anti-epileptogenic effect in a kainate-induced status epilepticus model.

In conclusion, we found that targeting a single cytokine, TNFα, can significantly delay the ictal development in OHSCs. Taken together, these data support the recent hypothesis that modulating specific pro-inflammatory mediators can provide new insights in anti-epileptic and anti-epileptogenic therapies [73]. Our experimental approach offers a unique and simultaneous access to various electrophysiological and molecular readouts which can be challenging in *in vivo* models thus providing a useful experimental tool for testing anti-inflammatory treatments and, more broadly, novel anti-epileptogenic therapies.

## Electronic Supplementary Material

ESM 1 (PDF 515 kb)

ESM 2 (PDF 506 kb)

ESM 3 (DOCX 146 kb)

ESM 4 (DOCX 137 kb)

ESM 5 (DOCX 125 kb)

ESM 6 (DOCX 140 kb)

ESM 7 (DOCX 88.1 kb)

ESM 8 (DOCX 101 kb)

ESM 9 (DOCX 16.1 kb)

ESM 10 (DOCX 15.8 kb)
