# Supplementary material for: Intrinsic Inflammation Is a Potential Anti-Epileptogenic Target in the Organotypic Hippocampal Slice Model
Source: Neurotherapeutics. 2018 Feb 20;15(2):470–88. doi: 10.1007/s13311-018-0607-6 (PMC5935638; doi:10.1007/s13311-018-0607-6)

**Supplementary Figure 1**

*Synchronized epileptiform activity across the hippocampal subregions*

Representative traces of epileptiform activity recorded for 30 min in CA1, CA3 and DG regions.


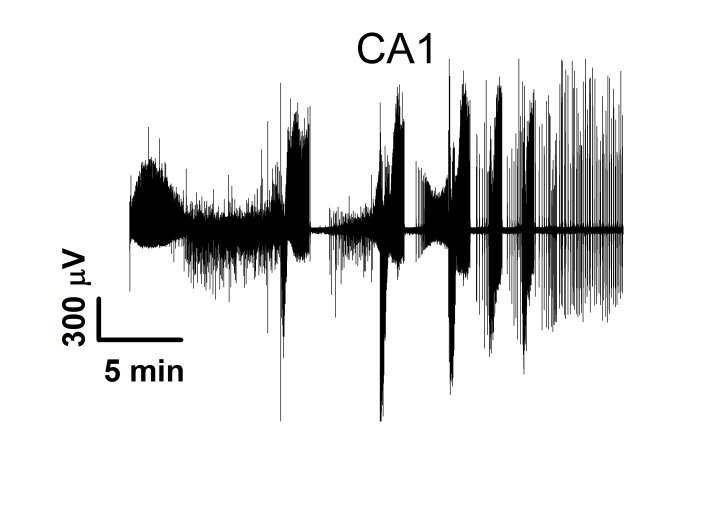

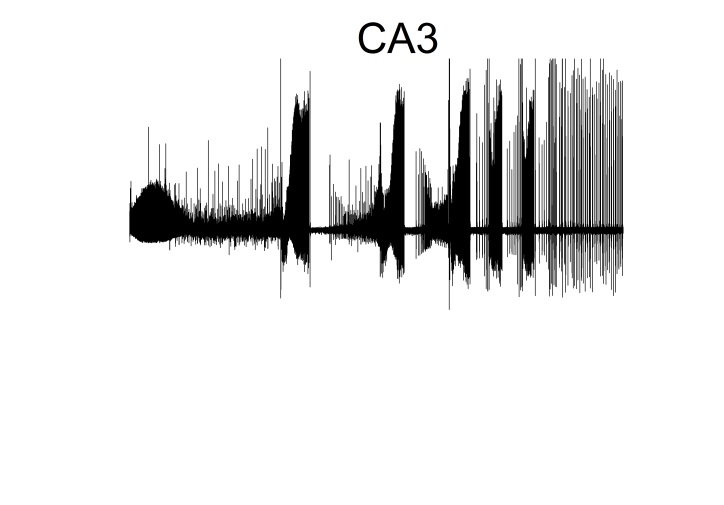

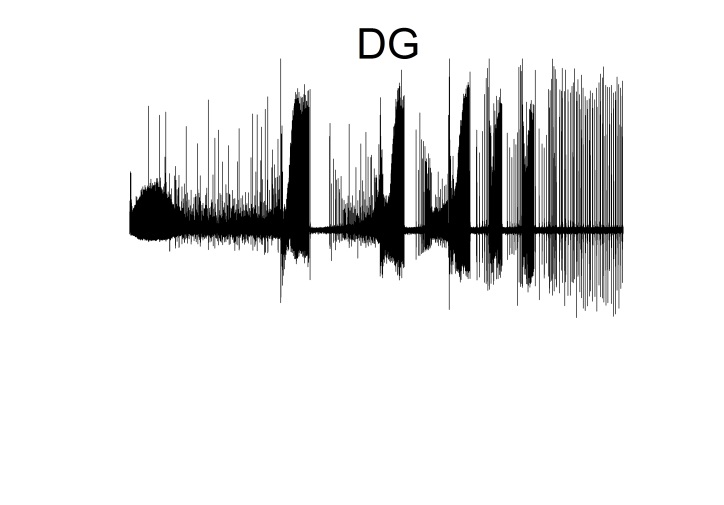

Supplement: Supplementary file 3 — (DOCX 146 kb) [file 13311_2018_607_MOESM3_ESM.docx]
