# Supplementary material for: Intrinsic Inflammation Is a Potential Anti-Epileptogenic Target in the Organotypic Hippocampal Slice Model
Source: Neurotherapeutics. 2018 Feb 20;15(2):470–88. doi: 10.1007/s13311-018-0607-6 (PMC5935638; doi:10.1007/s13311-018-0607-6)

**Supplementary Figure 2**

*Comparison of the cytokine levels at 11-13 DIV and 20 DIV*

The inflammatory cytokines IL-1β, TNFα and IL-6 levels at 11-13 DIV were compared with those at 20 DIV in slices cultured on MEAs and coverslips. * p<0.05 and *** p<0.001 by two-sample t-test. Data are presented as mean ± SEM.
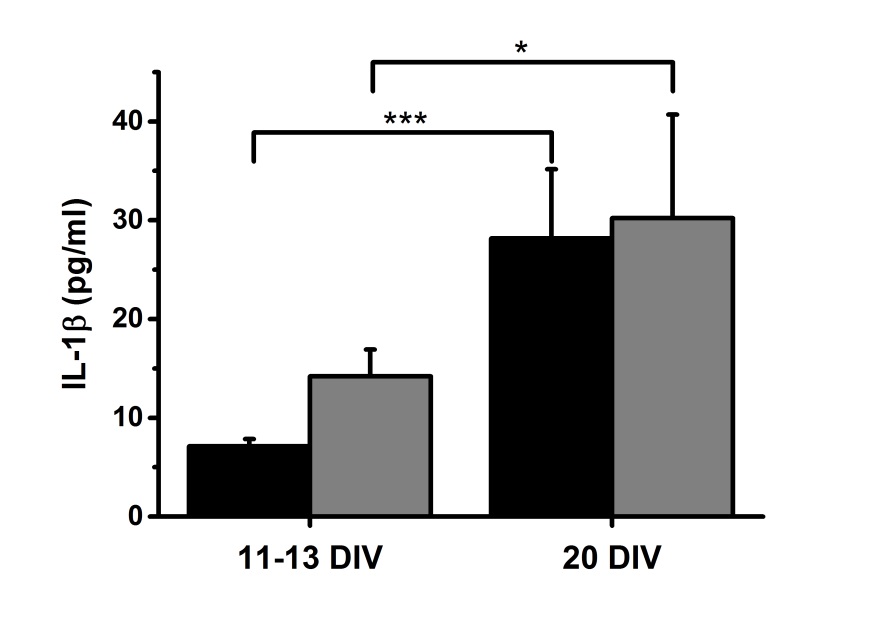

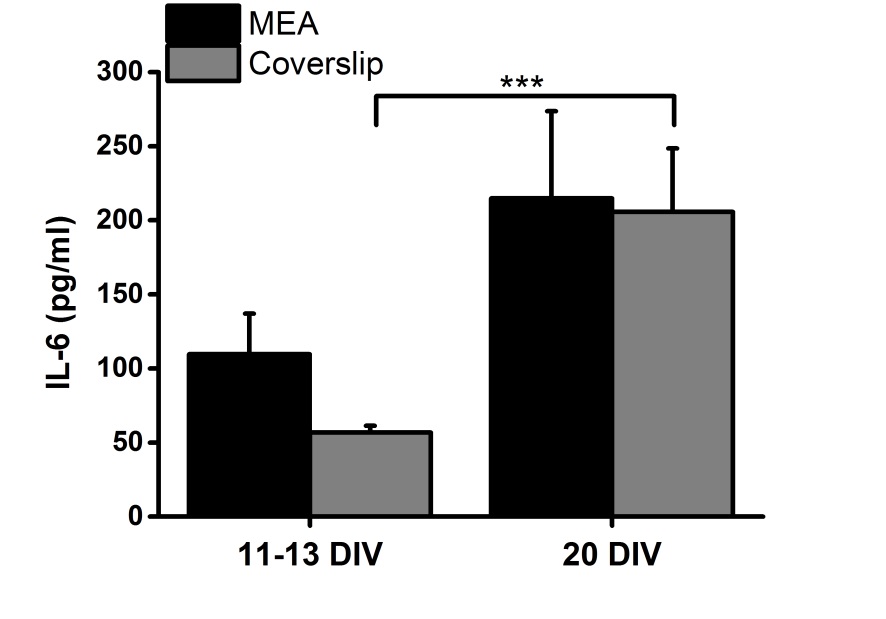

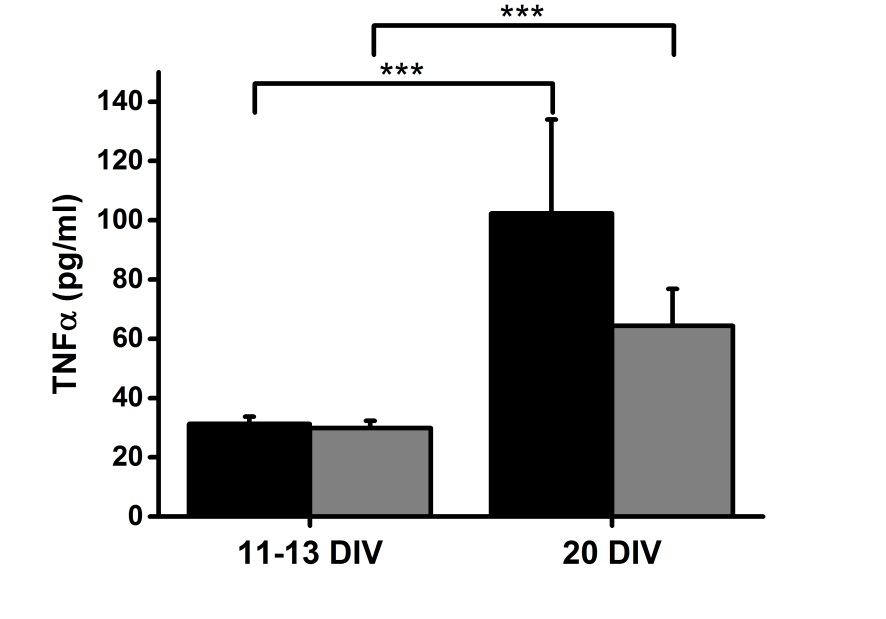

Supplement: Supplementary file 4 — (DOCX 137 kb) [file 13311_2018_607_MOESM4_ESM.docx]
