# Supplementary material for: Intrinsic Inflammation Is a Potential Anti-Epileptogenic Target in the Organotypic Hippocampal Slice Model
Source: Neurotherapeutics. 2018 Feb 20;15(2):470–88. doi: 10.1007/s13311-018-0607-6 (PMC5935638; doi:10.1007/s13311-018-0607-6)
Supplement: Supplementary file 5 — (DOCX 125 kb) [file 13311_2018_607_MOESM5_ESM.docx]

**Supplementary Figure 3**

*GFAP and CD11b staining quantification of hippocampal CA1, CA3 and DG hilus subfields at 3, 7, 14, 21 DIV*

Bargrams represent quantification of area positive with GFAP (A) and CD11b (B) staining expressed as percentage of the total area analyzed in hippocampal CA1, CA3 and DG hilus subfields in 3, 7, 14, 21 DIV OHSCs. n =4-6 slices/DIV. *p<0.05 and **p<0.01 vs 3 DIV; °p<0.05 and °°p< 0.01 vs 7 DIV; ^p<0.01 vs 14 DIV by one-way ANOVA followed by Tukey’s post-hoc test. Data are presented as mean ± SD. Statistical differences are among the same regions (CA1 vs. CA1; CA3 vs. CA3; Hilus vs. Hilus) at 3, 7, 14 and 21 DIV.
