# Supplementary material for: Intrinsic Inflammation Is a Potential Anti-Epileptogenic Target in the Organotypic Hippocampal Slice Model
Source: Neurotherapeutics. 2018 Feb 20;15(2):470–88. doi: 10.1007/s13311-018-0607-6 (PMC5935638; doi:10.1007/s13311-018-0607-6)
Supplement: Supplementary file 6 — (DOCX 140 kb) [file 13311_2018_607_MOESM6_ESM.docx]

**Supplementary Figure 4**

*Effect of anti-IL-6 polyclonal antibody on the development of ictal activity in OHSCs*

Incidence of ictal activity (A), mean number (B) and duration (C) of ictal were calculated and compared between anti-IL-6 and vehicle treated slices. No significant differences were observed. n=9-14 slices/treatment/DIV. Data are presented as mean ± SD.
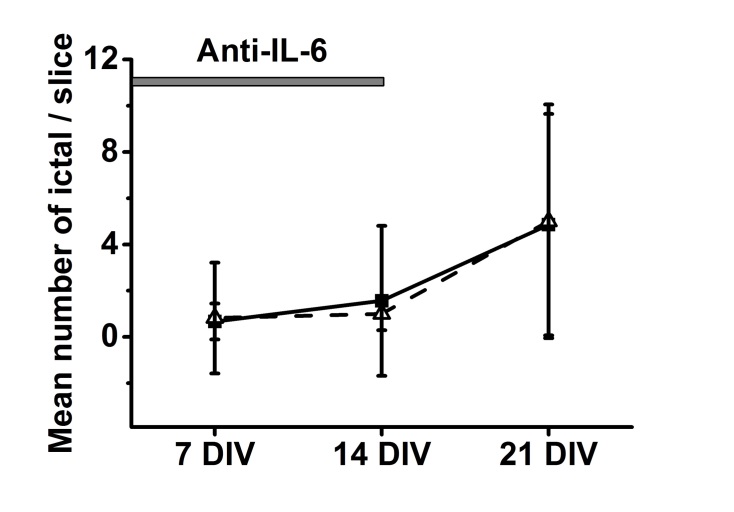

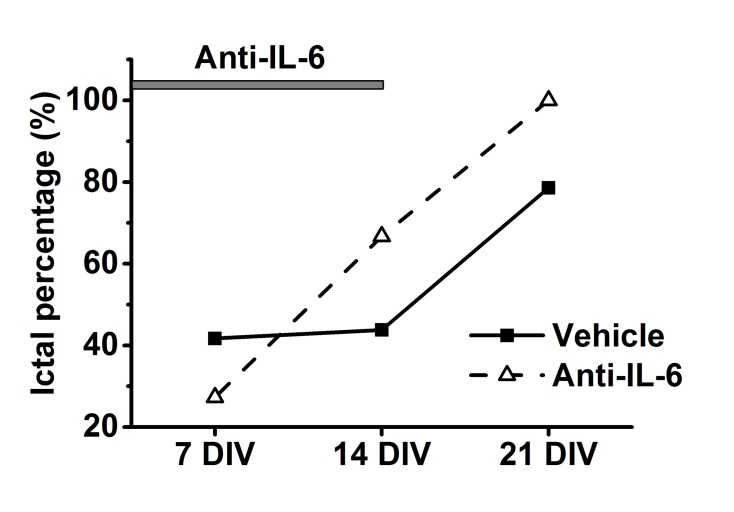

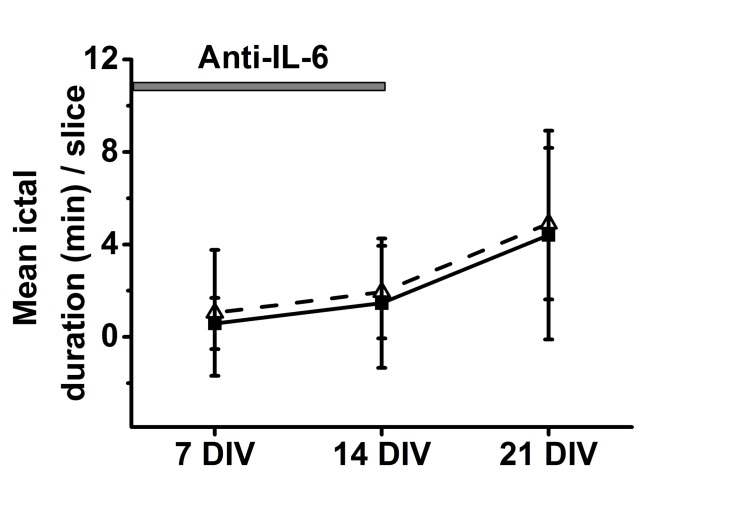


C

A

B
