# Supplementary material for: Intrinsic Inflammation Is a Potential Anti-Epileptogenic Target in the Organotypic Hippocampal Slice Model
Source: Neurotherapeutics. 2018 Feb 20;15(2):470–88. doi: 10.1007/s13311-018-0607-6 (PMC5935638; doi:10.1007/s13311-018-0607-6)

**Supplementary Figure 5**

*Anti-TNFα polyclonal antibody titration*

Polyclonal goat anti-TNFα antibody titration was measured by goat IgG ELISA kit from cultured supernatant collected at 14 (in the presence of the antibody) and at 16 and 20 DIV (after washout). Goat IgG was under the detection range in the vehicle group. n=9 slices for anti-TNFα group/DIV and 3 slices for vehicle group/DIV. Data are presented as mean ± SD.
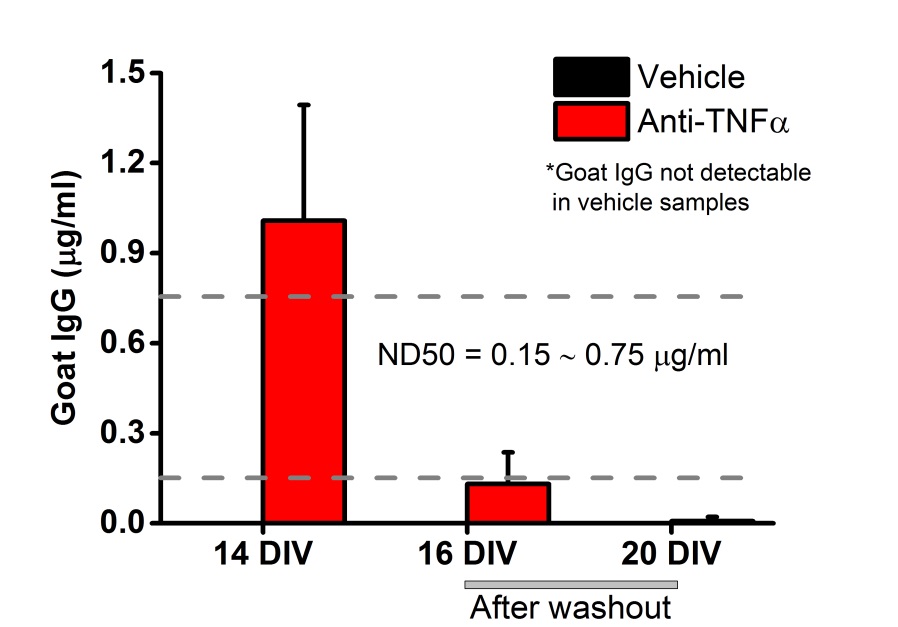

Supplement: Supplementary file 7 — (DOCX 88.1 kb) [file 13311_2018_607_MOESM7_ESM.docx]
