# Supplementary material for: Intrinsic Inflammation Is a Potential Anti-Epileptogenic Target in the Organotypic Hippocampal Slice Model
Source: Neurotherapeutics. 2018 Feb 20;15(2):470–88. doi: 10.1007/s13311-018-0607-6 (PMC5935638; doi:10.1007/s13311-018-0607-6)

**Supplementary Figure 6**

*Effect of anti-TNFα polyclonal antibody on the selected genes*

Principal component analysis (PCA) plot for 20 selected genes. PCA was performed from normalized mRNA expression levels measured by RT-qPCR. Each dot represents an individual slice culture sample collected at different time point in vehicle and anti-TNFα antibody group. n=6-10 slices/treatment/DIV.
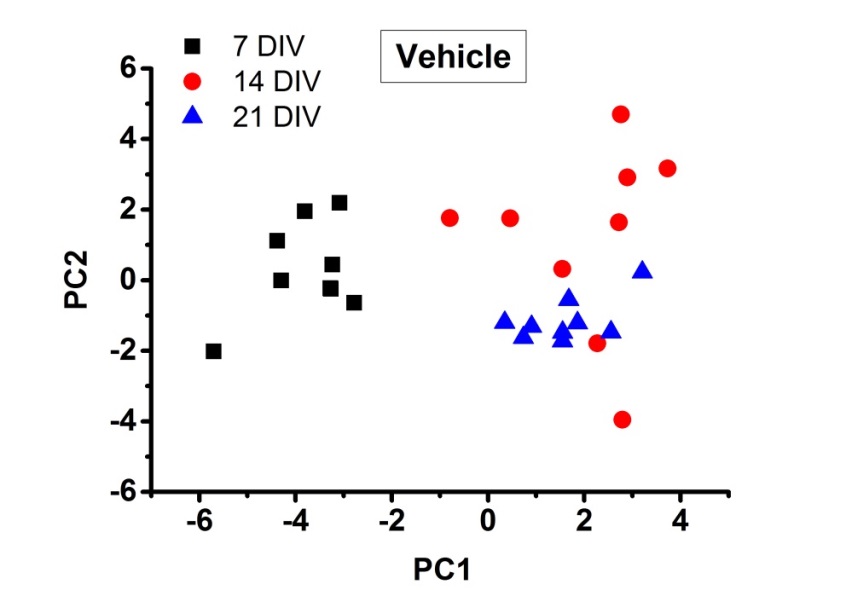

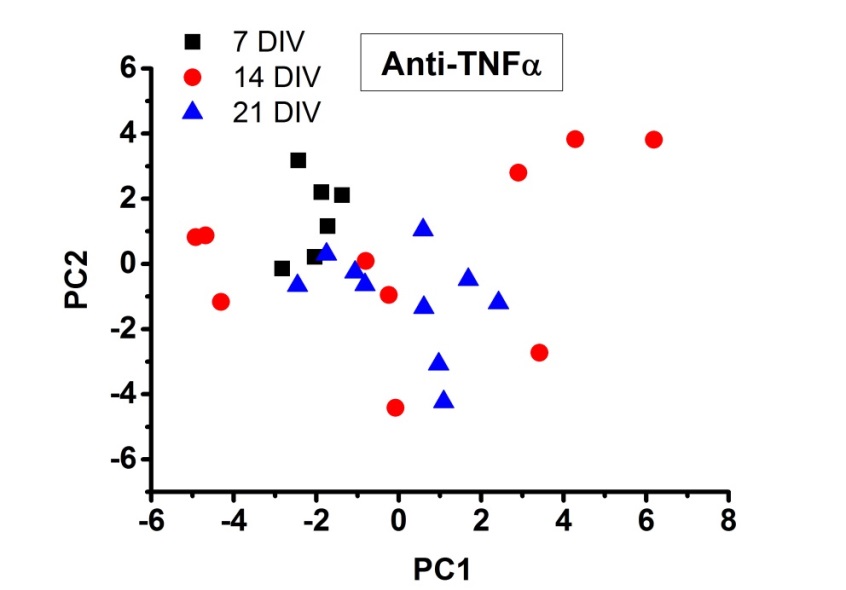

Supplement: Supplementary file 8 — (DOCX 101 kb) [file 13311_2018_607_MOESM8_ESM.docx]
