# Supplementary material for: Intrinsic Inflammation Is a Potential Anti-Epileptogenic Target in the Organotypic Hippocampal Slice Model
Source: Neurotherapeutics. 2018 Feb 20;15(2):470–88. doi: 10.1007/s13311-018-0607-6 (PMC5935638; doi:10.1007/s13311-018-0607-6)
Supplement: Supplementary file 9 — (DOCX 16.1 kb) [file 13311_2018_607_MOESM9_ESM.docx]

**Supplementary Table 1**

*Genes of interest*

| Gene name | Assay ID | Accession number |
| --- | --- | --- |
| Adam17 | Rn00571880_m1 | NM_020306.2 |
| Tradd | Rn01432142_g1 | NM_001100480.1 |
| Fadd | Rn00596168_m1 | NM_152937.2 |
| Casp8 | Rn00574069_m1 | NM_022277.1 |
| Traf-2 | Rn01758426_m1 | NM_001107815.2 |
| Rela (NFkB) | Rn01502266_m1 | NM_1999267.2 |
| Il6 | Rn01410330_m1 | NM_012589.2 |
| Il1 beta | Rn00580432_m1 | NM_031512.2 |
| GFAP | Rn00566603_M1 | NM_017009.2 |
| Itgam (Cd11b) | Rn00709342_m1 | NM_012711.1 |
| Birc2 | Rn01448127_m1 | NM_021752.2 |
| Jun | Rn99999045_s1 | NM_021835.3 |
| Casp3 | Rn00563902_m1 | NM_012922.2 |
| Map3K1 | Rn00588007_m1 | NM_053887.1 |
| RipK1 | Rn01757369_m1 | NM_001107350.1 |
| Iba1 (Aif1) | Rn00574125_g1 | NM_017196.3 |
| Ccl2 (Mcp-1) | Rn00580555_m1 | NM_031530.1 |
| Tnf | Rn01525859_g1 | NM_012675.3 |
| Tnfr1 | Rn01492348_m1 | NM_013091.1 |
| Tnfr2 | Rn00709830_m1 | NM_130426.4 |
| Ppib^$^ | Rn03302274_m1 | NM_022536 .2 |
| Hprt1^$^ | Rn01527840_m1 | NM_012583.2 |

^$^Ppib and hprt1 were used as reference genes
