# Supplementary material for: Intrinsic Inflammation Is a Potential Anti-Epileptogenic Target in the Organotypic Hippocampal Slice Model
Source: Neurotherapeutics. 2018 Feb 20;15(2):470–88. doi: 10.1007/s13311-018-0607-6 (PMC5935638; doi:10.1007/s13311-018-0607-6)
Supplement: Supplementary file 10 — (DOCX 15.8 kb) [file 13311_2018_607_MOESM10_ESM.docx]

**Supplementary Table 2**

*The list of genes whose expression levels show significant time-dependent increase in vehicle group when compared to anti-TNFα group*

| Gene | Vehicle | | | | Anti-TNFα | | | |
| --- | --- | --- | --- | --- | --- | --- | --- | --- |
|  | 7 DIV | 14 DIV | 21 DIV | p value | 7 DIV | 14 DIV | 21 DIV | p value |
| Fadd | 1.1 ± 0.1 | 1.6 ± 0.1 | 1.6 ± 0.1 | 5.03E-4*** | 1.5 ± 0.1 | 1.4 ± 0.1 | 1.6 ± 0.1 | 0.35 |
| Map3k1 | 1.0 ± 0.1 | 1.5 ± 0.1 | 1.3 ± 0.1 | 8.27E-5*** | 1.2 ± 0.1 | 1.3 ± 0.1 | 1.3 ± 0.1 | 0.69 |
| Casp8 | 1.0 ± 0.1 | 1.5 ± 0.1 | 1.8 ± 0.1 | 1.52E-5*** | 1.3 ± 0.2 | 1.3 ± 0.1 | 1.6 ± 0.1 | 0.13 |
| Adam17 | 1.0 ± 0.1 | 1.3 ± 0.1 | 1.3 ± 0.1 | 0.003** | 1.1 ± 0.1 | 1.2 ± 0.1 | 1.3 ± 0.1 | 0.15 |
| Tnfr1 | 1.0 ± 0.04 | 1.3 ± 0.04 | 1.3 ± 0.04 | 1.53E-4*** | 1.2 ± 0.1 | 1.2 ± 0.1 | 1.2 ± 0.04 | 0.88 |
| Tnfr2 | 1.0 ± 0.1 | 1.5 ± 0.1 | 1.4 ± 0.1 | 1.19E-4*** | 1.2 ± 0.1 | 1.1 ± 0.1 | 1.2 ± 0.1 | 0.82 |

Expression levels were normalized to the vehicle samples at 7 DIV (mean ± SEM). One-way ANOVA.
